# Factors Associated With Syphilis Transmission and Acquisition Among Men Who Have Sex With Men: Protocol for a Multisite Egocentric Network Study

**DOI:** 10.2196/40095

**Published:** 2022-11-04

**Authors:** Casey E Copen, Julie Rushmore, Alex De Voux, Robert D Kirkcaldy, Yetunde F Fakile, Carla Tilchin, Jessica Duchen, Jacky M Jennings, Morgan Spahnie, Abigail Norris Turner, William C Miller, Richard M Novak, John A Schneider, Andrew B Trotter, Kyle T Bernstein

**Affiliations:** 1 Division of STD Prevention National Center for HIV, Viral Hepatitis, STD and TB Prevention Centers for Disease Control and Prevention Atlanta, GA United States; 2 Division of Scientific Education and Professional Development Centers for Disease Control and Prevention Atlanta, GA United States; 3 Center for Child and Community Health Research Department of Pediatrics Johns Hopkins University School of Medicine Baltimore, MD United States; 4 Department of Health, Behavior and Society Bloomberg School of Public Health Johns Hopkins University Baltimore, MD United States; 5 Department of Epidemiology Bloomberg School of Public Health Johns Hopkins University Baltimore, MD United States; 6 Division of Epidemiology The Ohio State University Columbus, OH United States; 7 Department of Internal Medicine College of Medicine The Ohio State University Columbus, OH United States; 8 Division of Infectious Disease Department of Medicine University of Illinois College of Medicine Chicago, IL United States; 9 Departments of Medicine and Public Health Sciences University of Chicago Chicago, IL United States; 10 Howard Brown Health Chicago, IL United States

**Keywords:** sexually transmitted infection, HIV risk, men who have sex with men, sexual network, syphilis, mobile phone

## Abstract

**Background:**

In the United States, the rates of primary and secondary syphilis have increased more rapidly among men who have sex with men (MSM) than among any other subpopulation. Rising syphilis rates among MSM reflect changes in both individual behaviors and the role of sexual networks (eg, persons linked directly or indirectly by sexual contact) in the spread of the infection. Decades of research examined how sexual networks influence sexually transmitted infections (STIs) among MSM; however, few longitudinal data sources focusing on syphilis have collected network characteristics. The Centers for Disease Control and Prevention, in collaboration with 3 sites, enrolled a prospective cohort of MSM in 3 US cities to longitudinally study sexual behaviors and STIs, including HIV, for up to 24 months.

**Objective:**

The Network Epidemiology of Syphilis Transmission (NEST) study aimed to collect data on the factors related to syphilis transmission and acquisition among MSM.

**Methods:**

The NEST study was a prospective cohort study that enrolled 748 MSM in Baltimore, Maryland; Chicago, Illinois; and Columbus, Ohio. NEST recruitment used a combination of convenience sampling, venue-based recruitment, and respondent-driven sampling approaches. At quarterly visits, participants completed a behavioral questionnaire and were tested for syphilis, HIV, gonorrhea, and chlamydia. The participants also provided a list of their sexual partners and described their 3 most recent partners in greater detail.

**Results:**

The NEST participants were enrolled in the study from July 2018 to December 2021. At baseline, the mean age of the participants was 31.5 (SD 9.1) years. More than half (396/727. 54.5%) of the participants were non-Hispanic Black, 29.8% (217/727) were non-Hispanic White, and 8.8% (64/727) were Hispanic or Latino. Multiple recruitment strategies across the 3 study locations, including respondent-driven sampling, clinic referrals, flyers, and social media advertisements, strengthened NEST participation. Upon the completion of follow-up visits in March 2022, the mean number of visits per participant was 5.1 (SD 3.2; range 1-9) in Baltimore, 2.2 (SD 1.6; range 1-8) in Chicago, and 7.2 (SD 2.9; range 1-9) in Columbus. Using a community-based participatory research approach, site-specific staff were able to draw upon collaborations with local communities to address stigma concerning STIs, particularly syphilis, among potential NEST participants. Community-led efforts also provided a forum for staff to describe the NEST study objectives and plans for research dissemination to the target audience. Strategies to bolster data collection during the COVID-19 pandemic included telehealth visits (all sites) and adaptation to self-collection of STI specimens (Baltimore only).

**Conclusions:**

Data from NEST will be used to address important questions regarding individual and partnership-based sexual risk behaviors among MSM, with the goal of informing interventions to prevent syphilis in high-burden areas.

**International Registered Report Identifier (IRRID):**

RR1-10.2196/40095

## Introduction

### Background

Syphilis is a sexually transmitted infection (STI) caused by the bacterium *Treponema pallidum*. In the primary stage, syphilis spreads by direct contact with a syphilitic sore (ie, chancre), which can progress in the secondary stage to include symptoms such as skin rashes and mucous membrane lesions. In 2020, a total of 41,655 primary and secondary (P&S) syphilis cases were reported in the United States, and 81% of these cases were among men [1]. P&S syphilis rates have increased steadily among gay, bisexual, same-gender-loving, and other men who have sex with men (MSM) aged ≥15 years from 20.6 per 100,000 in 2000 to 272.8 per 100,000 in 2015 [2,3]. Both national and state-specific P&S syphilis rates are higher among MSM than among men who have sex with women only (309 per 100,000 compared with 2.9 per 100,000 in 2015) [4]. In 2020, MSM accounted for 43% of reported P&S syphilis cases, and nearly half of MSM with P&S syphilis in 2020 were also living with HIV [5]. HIV and syphilis are often linked because syphilis can cause inflammatory genital ulcers and lesions, which can increase the risk of HIV transmission [6-9].

Several network factors have been implicated in the rise of syphilis cases among MSM, including increase in condomless anal sex and multiple or concurrent (ie, overlapping) sexual partners [10-13]. MSM are more likely to report having concurrent sexual partnerships than heterosexual individuals [14]. Concurrent partnerships can create larger, more connected sexual networks that can amplify the spread of STIs [15-19]. Biomedical HIV interventions such as pre-exposure prophylaxis (PrEP) to prevent HIV infection and antiretroviral therapy for persons living with HIV can also affect STI risk within networks. The selection of partners who use these modalities can affect condom use and serosorting behaviors (ie, limiting partners to same HIV status) both within and across partnerships [20-22]. Racial homophily (ie, same race or ethnicity partners) is another network factor that can affect syphilis case rates, particularly within MSM networks where syphilis and HIV are concentrated [23-28]. The influence of racial homophily on syphilis rates within sexual networks among MSM can be exacerbated by social and environmental factors, such as racism, segregation, poverty and income inequality, education, stigma, discrimination, and access to health care [29-31].

Although research has examined individual and network-related factors contributing to rising syphilis case rates among MSM [32-35], most studies are cross-sectional and cannot account for how individual STI or HIV risk may vary as partnerships change. For example, specific cycles of the National HIV Behavioral Surveillance that focus on MSM sexual behaviors confine questions on sexual partnerships to a 12-month retrospective reference period [36]. Similarly, case reports from disease intervention specialists from local health departments are limited to patients who test positive for STI or HIV, and partner-level information is typically collected only when the partner can be located for STI testing or treatment [37]. Individuals’ risk of syphilis acquisition and transmission may change over time depending on the behaviors enacted during specific sexual encounters, such as the introduction of new sex partners. Similarly, the context of these new and continuing relationships may affect sexual behavior (eg, condom use and types of sex [oral or anal]). To fill gaps in what is currently known about syphilis epidemiology among MSM, the Centers for Disease Control and Prevention (CDC) partnered with 3 sites to conduct the Network Epidemiology of Syphilis Transmission (NEST) study.

### This Study

NEST was a prospective, longitudinal cohort study aimed to identify drivers of change in syphilis epidemiology and account for network factors affecting syphilis transmission dynamics among MSM. NEST included MSM from the Mid-Atlantic (Baltimore City, Maryland) and Midwestern (Chicago, Illinois, and Columbus, Ohio) regions of the United States. Each of these geographic areas has rates of syphilis among MSM that are higher than the national rate [1,38-41]. In addition, the associated counties were among those identified as *hotspots*, where >50% of the total new HIV diagnoses occurred from 2016 to 2017 [42]. This paper details the NEST research protocol, the impact of the novel COVID-19 pandemic on NEST data collection, and the successes and challenges in collecting sexual health information among MSM in these geographic areas.

## Methods

### Study Design

NEST was designed and implemented by Johns Hopkins University in partnership with the Baltimore City Health Department, University of Illinois at Chicago (in partnership with Howard Brown Health), and Ohio State University. NEST study participants completed a structured questionnaire about their sexual and health behaviors over time and provided biological samples at each study visit to test for syphilis, HIV, gonorrhea, and chlamydia. The NEST self-administered survey took approximately 60-90 minutes to complete. During the course of the study, participants received US $40-75 for participating in the survey and diagnostic STI testing and US $10-25 for referring each eligible person that was part of their social or sexual network.

### Consent to Participate

The participants provided written informed consent before enrolling. The study staff conducted surveillance activities in adherence to ethical principles and standards by respecting and protecting the privacy, confidentiality, and autonomy of the participants to the maximum extent possible.

### Ethics Approval

This study obtained oversight from the CDC institutional review board (IRB) and approval from local institutional IRBs at Johns Hopkins University, the University of Illinois at Chicago, and Ohio State University.

### Participant Selection and Recruitment

Data collection for NEST was conducted from July 2018 to March 2022. Participants were followed for up to 24 months with quarterly study visits. Participants were recruited with assistance from local health departments, health clinics, and community-based organizations using multiple recruitment strategies, including convenience sampling, venue-based recruitment, and respondent-driven sampling (RDS) techniques. The recruitment strategies and target populations for NEST were tailored according to the local epidemiology of syphilis among MSM at each study site. The study’s inclusion criteria and tailored recruitment strategies are presented in Textbox 1.

**Inclusion criteria and tailored recruitment strategies.**

**Inclusion criteria**

- Assigned male sex at birth
- Currently identifies as male
- Aged ≥18 years
- Had oral or anal sex with a man in the 6 months before baseline interview
- Lived within the study area
- Able to complete the survey in English
- Provided written informed consent

**Tailored recruitment strategies (implementation varied across study sites)**

- Aged ≤45 years
- Diagnosed with a new, untreated, or recent syphilis infection (6 months before the baseline interview, regardless of treatment)

### Formative Research Phase

During the formative research phase of the study (ie, 1 year before study enrollment), NEST investigators focused on developing research methods that were transparent and culturally appropriate and that collected information that could be readily applied to local interventions. A community-engaged participatory approach that included community advisory boards (CABs) and focus groups was used to build trust between researchers and participants and to identify ways in which study participation would benefit the broader community [43,44]. In Baltimore, the CAB consisted of 6 to 7 Black MSM who met monthly throughout the study period. Examples of CAB activities included codeveloping new survey questions, providing feedback on community dissemination materials, and designing a substudy within NEST. In addition, based on needs identified via qualitative data gathered during the formative period, Baltimore strengthened the capacity of community members hired as project staff through education, training, and employment and routinely shared interim findings with NEST participants, relevant community-based organizations, and partnering clinical and nonclinical sites [43].

### Questionnaires

#### Individual-Level Measures

Participants were asked about sociodemographic and health-related information in self-administered surveys. The sociodemographic characteristics included age, race and ethnicity, sexual orientation, education, employment status, and relationship status (refer to individual characteristics in Textbox 2). To describe health care–seeking behavior and prior STIs, participants were asked about their health insurance status, where they usually received STI-related health care, HIV status, syphilis diagnoses (lifetime and in the past 12 months), prior gonorrhea and chlamydia diagnoses (including anatomical site), PrEP knowledge, PrEP prescription and use, and frequency of STI or HIV testing (refer to the health care access and use section in Textbox 2). In addition, participants were asked about individual-level sexual behaviors, including the number of sex partners in the past 3 months, types of sex, drug or alcohol use during sex, experience of exchange sex, and group sex (refer to individual sexual behavior [in the past 3 months] in Textbox 2). The types of sex (giving or receiving) that participants were asked about included anal sex, oral sex (ie, oral-genital), rimming (ie, oral-anal), and vaginal sex. Specific information on these topics, as well as other sex partner characteristics, was collected about the 3 most recent partners in the past 3 months, including those partners’ HIV status and use of PrEP (refer to sexual behavior with 3 most recent sex partners [in the past 3 months] and partner characteristics [in the past 3 months] in Textbox 2). The interview concluded with questions about syphilis symptoms (refer to syphilis symptoms in Textbox 2), substance use (refer to substance use in Textbox 2), and PrEP attitudes and practices (refer to HIV PrEP in Textbox 2).

**Survey domains in the Network Epidemiology of Syphilis Transmission study.**

**Individual characteristics**

- Age
- Race or ethnicity
- Sexual orientation
- Sexual role (eg, “top” [insertive partner], “bottom” [receptive partner], or “versatile” [either insertive or receptive partner])
- Education or employment status
- Relationship status
- Experience with homelessness, food insecurity, and prison or jail

**Health care access and use**

- Current health insurance
- Type of health insurance
- Time since last saw a physician, nurse, or health care provider
- Inability to afford medical care
- Sexually transmitted infection (STI) testing in the past 12 months
- Regular place for STI-related health care
- HIV testing (ever or in the past 12 months)
- Last HIV test result
- Self-reported history of syphilis, gonorrhea, and chlamydia diagnoses
- HIV pre-exposure prophylaxis (PrEP) knowledge, prescription, and use
- HIV postexposure prophylaxis use

**Individual sexual behavior (in the past 3 months)**

- Number of sex partners
- Types of sex (oral-genital, anal, oral-anal, or vaginal)
- Drug or alcohol use before or during sex
- Exchanging sex for money, drugs, shelter, or something else
- Group sex

**Sexual behavior with 3 most recent sex partners (in the past 3 months)**

- Types of sex (oral-genital, anal, oral-anal, or vaginal)
- Frequency of sex and date of sex
- Condom use at last sex (oral-genital, anal, oral-anal, or vaginal)
- Exchanging sex for money, drugs, shelter, or something else
- Sex partner had concurrent (ie, overlapping) sex partners

**Partner characteristics (in the past 3 months)**

- Name or nickname
- Sex assigned at birth
- Gender identity (ie, personal sense of one’s own gender)
- Age and race or ethnicity
- Sexual relationship (main or casual)
- Where they first met
- Partners’ HIV status
- Partners’ antiretroviral status
- Partners’ PrEP use

**Syphilis symptoms**

- Signs and symptoms related to syphilis

**Substance use**

- Injection drug use
- Noninjection drug use
- Alcohol use

**HIV PrEP**

- HIV PrEP knowledge, attitudes, and practices

#### Sexual Network Data Collection

In the interviewer-led portion of the interview, participants were asked to recount all sexual partnerships in the past 3 months, provide basic demographic information about these partners, and provide detailed characteristics for the 3 most recent sex partners. Participants were asked to provide the first and last name, first name and last initial, or a nickname for each partner so that partners could be consistently identified at quarterly visits.

All partners named at the baseline visit were considered a new sex partner. For each partner named by the participant at subsequent visits, the interviewer checked the cumulative list of previously named partners to determine whether this partner was named for the first time or was named at a previous visit (eg, “You have not told me about [new partner name] before. Is that correct?”). After the participant free-listed the names of their partners from the past 3 months (ie, without interviewer prompting), the interviewer would confirm whether any partners not renamed from the previous visit were still current sex partners (eg, “At the last visit you told me about [old partner name]. Have you had sex with this partner in the last 3 months?”).

**Figure 1:**
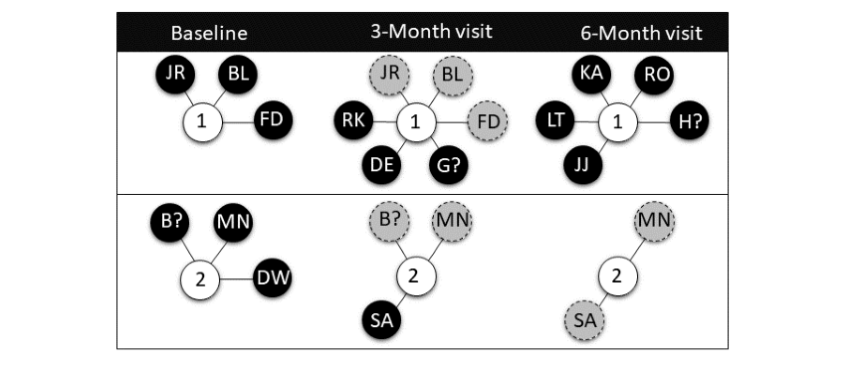
Hypothetical sociogram of 2 Network Epidemiology of Syphilis Transmission study participants at the baseline and 3-month and 6-month study visits (black circles represent named partners, gray circles represent renamed partners; the letters within circles represent initials provided by participants; the question marks represent names or identifiers that may not be known).

Figure 1 illustrates a hypothetical sociogram (ie, graphical representation of interpersonal relationships) for 2 participants at the baseline and 3-month and 6-month study visits. This figure presents an example of the complexity of sexual relationships that can exist among study participants [45]. Participants 1 and 2 both reported the initials of 3 sex partners at their baseline study visit. Participant 1 reported retaining all 3 baseline partners (“JR,” “BL,” and “FD”) in addition to acquiring 3 new partners (“RK,” “DE,” and “G?”) at the 3-month visit. The question mark indicates names or identifiers that may not be known. At the 6-month visit, participant 1 reported 5 new partners (“KA,” “RO,” “H?,” “JJ,” and “LT”), with no previous partners retained. At the 3-month visit, participant 2 reported retaining 2 baseline partners (“B?” and “MN”) and acquiring 1 new partner (“SA”) at the 3-month visit. At the 6-month visit, participant 2 reported retaining 2 partners (“SA” and “MN”) with no new partners.

### Biological Samples Collection

#### Overview

Consenting participants provided biological samples for STI or HIV screening at baseline and at each quarterly visit. Positive STI or HIV results were reported to the local health department according to the state and local reporting requirements. Participants with positive test results were referred to the health department or the provider of their choice for treatment.

#### HIV Sample

Trained HIV counselors collected blood samples for rapid HIV testing (eg, INSTI antibody test) or fourth-generation enzyme immunoassay. Confirmatory testing (eg, polymerase chain reaction for HIV-1) was conducted on all individuals who tested positive for HIV.

#### Syphilis

Trained phlebotomists collected 2 blood samples: one sample for testing at a local clinic where results were returned to participants and a second sample for additional testing at the CDC for evaluation of syphilis diagnostic algorithms and longitudinal monitoring of serum antibody titers in incident and repeat syphilis infections. A sequential combination of nontreponemal tests (most often the rapid plasma reagin) and treponemal tests (eg, fluorescent treponemal antibody test absorption test, among others) was used locally to screen samples for *T. pallidum*.

#### Gonorrhea and Chlamydia

Urethral, anorectal, and oropharyngeal swabs were either self-collected or collected by the clinical staff, depending on the local protocol. Nucleic acid amplification tests were performed locally to test samples for the genetic material of *Neisseria gonorrhoeae* and *Chlamydia trachomatis*.

### Ecological Momentary Assessment

A subsample of NEST participants agreed to participate in the collection of supplemental data with an ecological momentary assessment (EMA). EMA was a smartphone app comprising short questions to capture the occurrence of specific sexual behaviors in real time. Participants used their own smartphone, or were provided with a smartphone, to respond to brief surveys each day for a 2-week period. In Baltimore, the EMA was augmented with geolocation data (eg, a GPS data point) that was collected while the phone was turned on with a cell phone service [46]. Survey questions included the number of people with whom the participant had oral or anal sex in the past 24 hours, where the participant met sex partners, where sex took place, use of mobile geosocial networking apps to meet sex partners (eg, Grindr, Manhunt, among others), and drug or alcohol use. Data from Baltimore on EMA participation indicated that those who participated were willing to complete the brief surveys and answer the questions when prompted [46].

### Data Management

Each study site maintained a centralized data management system for NEST data using their own software (eg, REDCap [Research Electronic Data Capture], Vanderbilt University; Qualtrics, Qualtrics XM) [47,48]. The NEST study participants were assigned a unique study ID number for all study documents and biospecimens. At each study visit, interviewers entered a unique identifier for all sex partners in the past 3 months in the data entry form that corresponded with the participants’ study records. The study record was retained throughout the study if the partner was mentioned at subsequent visits. To maintain confidentiality, personally identifiable information was removed from the data before transmission to the CDC’s Division of Sexually Transmitted Disease Prevention (DSTDP).

To ensure consistency across study sites, NEST staff from the DSTDP’s Surveillance and Data Science Branch provided a data dictionary that contained the questions and variable text, variable names, field limits, skip logic, consistency checks, response values, and formats. Data managers within each site worked with DSTDP to transmit data in a standardized format via a secure application on a quarterly schedule. The CDC’s DSTDP NEST staff was responsible for the accuracy, quality, completeness, and internal consistency of the NEST data.

### Adaptations to Data Collection During the COVID-19 Pandemic

Beginning in March 2020, the COVID-19 pandemic introduced challenges for NEST study operations as well as partnering state and local public health programs, including closures of clinics, universities, and health departments. During the early months of the pandemic, NEST study sites rapidly transitioned from in-person visits to remote-only visits, in which interviews were conducted via telephone or on the web. During the early months of the pandemic, study staff in Baltimore and Columbus were often successful in scheduling follow-up calls with participants while shelter-in-place orders were in effect. Participants who were already familiar with the questions asked during visits often came prepared by bringing a list of their sexual partners. However, telehealth approaches did not work as effectively in Chicago, where challenges included the limited ability to locate or reach participants who did not have reliable internet or mobile phone services or who experienced housing instability.

The COVID-19 pandemic presented challenges for in-person clinical visits in all 3 NEST sites. In both Chicago and Columbus, STI testing for chlamydia, gonorrhea, HIV, and syphilis was halted in March 2020 for approximately 8 months. Baltimore had the opportunity to provide 2 innovative methods for STI specimen self-collection (ie, not under medical supervision) to its participants. Baltimore participants were offered mail-in testing of self-collected samples through (1) an existing web-based outreach program (“I Want the Kit” [IWTK] [49]) and (2) Molecular Testing Labs (MTL), a provider of diagnostic testing. Historically, IWTK has had a high level of acceptability among its users [50]. Through a specific arrangement with IWTK, chlamydia, gonorrhea, and HIV testing was offered to participants in Baltimore from June 2020 onward. Approximately 200 Baltimore NEST participants enrolled in the participating clinics were sent IWTK collection kits, and of these, 81.5% (163/200) returned the kits for testing.

From January 2021 onward, Baltimore began using MTL for chlamydia, gonorrhea, HIV, and syphilis testing. Syphilis testing was performed using a dried blood spot treponemal test kit. Participants were asked to self-collect 5 to 10 drops of blood from a fingerstick on a blood collection card. Approximately 71 participants in Baltimore with no prior positive nontreponemal test were sent collection kits from MTL and 66% (47/71) sent the kits back to MTL for syphilis antibody testing.

For STI tests collected through IWTK and MTL, positive results were reported to the Baltimore City Health Department where disease investigation specialists notified and referred participants for treatment via standard health department protocol. The availability of STI test results outside of an in-person visit was important for timely access to treatment during a time of reduced clinic operations. Some Baltimore participants experienced challenges using STI specimen self-collection methods, including United States Postal Service delays in kit deliveries, lags in the receipt of testing results, and delays in remuneration after receiving results. Furthermore, some participants who self-collected fingerstick blood for syphilis testing via MTL reported challenges with the procedure, such as difficulty puncturing fingers and problems following the instructions for blood sample self-collection, which could lead to reduced specimen quality. Despite the potential barriers, the high uptake of these STI self-collection strategies among NEST participants in Baltimore adds to the existing studies on the feasibility and acceptability of such programs among MSM in other jurisdictions [51].

### Planned Analyses

CDC and NEST-affiliated researchers are using network analysis approaches to understand how individual-level behaviors and network-level factors affect the acquisition of syphilis and other STIs. These methodological approaches include estimating and simulating sexual network models with respect to the longitudinal fluctuations in partnerships and STI transmission dynamics. Other analyses will explore factors associated with partnership characteristics, partnership duration and turnover, and STI incidence among participants. We will also explore longitudinal changes in sexual behavior during the COVID-19 pandemic.

## Results

NEST enrollment began on July 20, 2018 and concluded on December 17, 2021; a total of 748 MSM (444/748, 59.3%, in Baltimore; 63/748, 8.4%, in Chicago; and 241/748, 32.2%, in Columbus) were enrolled. Enrollment ended in March 2020 for Baltimore and Columbus and in December 2021 for Chicago. The mean age of the participants was 31.5 (SD 9.1) years (Table 1), and it ranged from 18-45 years in Baltimore, 21-56 years in Chicago, and 18-77 years in Columbus. The average number of visits per participant was 5.1 (SD 3.2; range 1-9) in Baltimore, 2.2 (SD 1.6; range 1-8) in Chicago, and 7.2 (SD 2.9; range 1-9) in Columbus. Overall, more than half (396/727, 54.4%) of the participants were non-Hispanic Black, 29.8% (217/727) were non-Hispanic White, and 8.8% (64/727) were Hispanic or Latino. Approximately 64.9% (485/747) of the participants had some college education or a bachelor’s degree. Most (558/747, 74.7%) participants identified as gay and 19.5% (146/747) identified as bisexual. Most (586/704, 83.2%) participants had private or public health insurance and 67.5% (504/747) were currently employed. When asked about the previous 6 months, 21.2% (158/746) of the participants reported experiencing homelessness (ie, no regular place to stay for at least one night), and 43.2% (323/745) of the participants experienced food insecurity. Approximately 7.6% (57/747) of the participants reported prison or jail experience in the past 12 months.

The demographic characteristics of NEST participants at baseline from Baltimore, Chicago, and Columbus describe a racially or ethnically diverse group of MSM, most of whom had at least some college education, full- or part-time employment, and health insurance. Most of the MSM participants identified as gay, but 24.5% (183/747) of the participants reported other sexual identities (ie, bisexual or “something else”). Potential differences in sexual behaviors by sexual orientation may have implications for corresponding STI risk [52]. Participants’ experiences of recent housing instability or food insecurity are examples of social determinants of health that could contribute to disparities in STI diagnoses among MSM [53,54].

**Table 1:** Baseline characteristics of men who have sex with men participating in the Network Epidemiology of Syphilis Transmission study (N=748).

Characteristics				Values^a^
Age (years), mean (SD)				31.5 (9.1)
**Hispanic origin or race (n=727), n (%)**				
	Hispanic or Latino		64 (8.8)	
	Non-Hispanic Black		396 (54.5)	
	Non-Hispanic White		217 (29.8)	
	Non-Hispanic other or multiple race		50 (6.9)	
**Education (n=747), n (%)**				
	Less than high school		54 (7.2)	
	High school or general equivalency diploma		208 (27.8)	
	Some college		239 (32)	
	Bachelor’s degree or higher		246 (32.9)	
**Sexual orientation (n=747), n (%)**				
	Gay		558 (74.7)	
	Straight		6 (0.8)	
	Bisexual		146 (19.5)	
	Something else		37 (5)	
**Current health insurance status^b^ (n=704), n (%)**				
	Insured		586 (83.2)	
	Uninsured		118 (16.8)	
**Employment status^c^ (n=747), n (%)**				
	Employed		504 (67.4)	
	Not employed		243 (32.5)	
**Past 6 months, n (%)**				
	**Homelessness^d^ (n=746)**			
		Yes	158 (21.2)	
		No	588 (78.8)	
	**Food insecurity (n=745)**			
		Yes	323 (43.3)	
		No	422 (56.6)	
**Past 12 months, n (%)**				
	**Prison or jail experience (n=747)**			
		Yes	57 (7.6)	
		No	690 (92.3)	

^a^Percentages may not add to 100% due to rounding.

^b^Insurance status included public or private insurance.

^c^Employed: full-time work, part-time work, self-employed, or studying; not employed: unemployed, retired, stay-at-home parent, or unable to work.

^d^Homeless was defined as “living on the street, in a shelter, in a single room occupancy (SRO) hotel, with friends, in a car, or you have not had a regular place to stay for at least one night.”

## Discussion

### Significance of the Study

The NEST study is unique in its assessment of individual- and partner-level characteristics in MSM networks using quarterly behavioral surveys and repeated STI or HIV testing. The detailed list of sexual partners and partner characteristics collected at each interview provides important information about relationship dynamics and concurrency that could be an indication of the participants’ connection to a larger sexual network. Public health surveillance data in Baltimore, Chicago, and Columbus have documented rising syphilis rates among MSM [38-41]. In addition to individual-level factors that could affect syphilis case rates, syphilis factors at the network level include injection drug use and party drug use [55] and the use of geospatial network applications to meet sex partners on the web [55-57]. The results of this study may identify local drivers of syphilis transmission that can assist health departments in targeting limited resources (eg, disease intervention specialist interviews) and developing tailored syphilis screening programs among populations of interest. Additional analyses described in the Planned Analyses section are underway with analyses and dissemination of results expected to continue through 2024.

### Lessons Learned

Here, we summarize the lessons learned in the hope that future research studies among MSM using a prospective egocentric network study design can use this information to facilitate recruitment and enhance data quality. We focus the discussion specifically on the factors associated with NEST recruitment and sexual network data collection as well as how the study addressed STI stigma, specifically regarding syphilis, among participants.

### NEST Recruitment Strategies

Each study site developed recruitment procedures that were guided by consultations with local CABs on the best practices for community engagement. Recruitment procedures included RDS; flyers at relevant locations; targeted community events; partnerships with HIV testing agencies; clinics, hospitals, and community-based organizations; and social media (eg, by placing advertisements on Facebook and dating apps such as Grindr). For RDS, each seed was given coupons to disperse to their sexual or social network peers. RDS was kickstarted at each site during the formative phase of NEST (ie, before pandemic) when in-person meetings could be held to introduce the study to the local community. Approximately 54% (34/63) of the participants in Chicago, 36.9% (164/444) of the participants in Baltimore, and 24.9% (60/241) of the participants in Columbus were recruited through RDS. For Chicago, RDS was the primary method for study recruitment; however, this method proved challenging to implement because the participants’ coupons were often not distributed, and many coupon recipients could not be located or were ineligible for the study.

Recruitment methods across sites evolved based on initial enrollment. The RDS recruitment method was adapted over the course of the study in Chicago. Initially, only seeds with a new, untreated diagnosis of syphilis were recruited. To include a larger sample of MSM, eligibility criteria were later expanded to include seeds with a history of syphilis in the last 6 months. In contrast, Baltimore started with broader eligibility criteria, which were narrowed during the study to identify more potential participants with a recent syphilis infection. These eligibility changes were approved by the local IRBs.

### Missing and Truncated Sexual Network Data

In addition to the self-administered survey, a central task of NEST data collection was for participants to provide a list of their sexual partners’ names, nicknames, or aliases and to describe the demographic (ie, for all partners) and behavioral characteristics (ie, for 3 most recent sex partners) to an interviewer. Whether occurring in person or remotely during the COVID-19 pandemic, this face-to-face activity may have been onerous for some participants who had difficulty recalling such details accurately. Furthermore, participants may not have wanted to disclose behaviors to an interviewer because of social desirability concerns, such as anonymous sexual partners and the sensitive nature of questions about recent sexual behaviors [58].

To enhance data quality, study sites relied heavily on staff training of network elicitation methods and use of the appropriate software. However, as is common with egocentric network studies, there may have been a truncation of partnership data when interviewers asked participants about the characteristics of recent sex partners (eg, last 3 partners in the past 3 months) [59,60]. Participants may have prioritized reporting partners that they knew more about and omitted those for whom they had less information. The biases associated with methods of partner recall in egocentric network studies are important to consider when studying STI or HIV transmission. When designing a sexual network study, investigators can compare the NEST study methods presented here with other methods for partner elicitation [61,62] to determine the best practices for describing network connectivity when relying on participants’ retrospective reports.

### STI-Related Stigma

STI-related stigma among MSM hinders the disclosure of potential STI exposure and symptoms to health care providers, which may influence timely STI testing and treatment [63,64]. Medical mistrust can also be a barrier for routine sexual health care engagement, particularly among Black MSM given their historical and present experiences with racism in medicine, especially syphilis care [65]. Prior qualitative work has shown that syphilis has a stronger stigma among MSM than HIV [66]. In some sites, potential participants reported reluctance to enroll in NEST because of the study’s emphasis on syphilis testing. To address these concerns, site-specific staff used participatory research techniques to build trust with Black MSM and provide a forum for researchers and the community to interact and exchange information about the study [43,66].

Similar efforts to reduce stigma associated with syphilis included the development of forward-facing materials for NEST, such as rebranding the study name for local websites, and using local community members as models for advertisements and flyers [67]. For example, in Baltimore, the NEST study was named USHINE (“Understanding Sexual Health in Networks”) to emphasize the study’s broader focus on sexual health issues rather than on syphilis specifically. Study sites are evaluating locally how these recruitment and enrollment materials affected study recruitment and retention to inform future network studies involving MSM.

### Dissemination of Study Findings

NEST study sites used a community-based participatory research approach to inform all research activities, including the development of a research dissemination strategy. For example, Baltimore’s NEST study, USHINE, worked with CAB members to develop a plan to share results with participants, including share-back events and informational cards given to participants (web-based during COVID-19) after each study visit. The CAB members emphasized ways to share sexual health messages on these study follow-up cards, which included medical information about syphilis, other sexual health factors specific to the community (eg, syphilis rates), local resources, and events of interest. In addition to the study follow-up cards, CAB members encouraged study staff to use social media to communicate the results of the study and to promote local sexual health resources.

### Conclusions

NEST data provide a unique opportunity to understand changes in sexual behaviors and sexual networks among MSM participants, particularly as STI case rates and STI or HIV prevention and care activities continue to be affected by COVID-19. The individual- and network-related characteristics of MSM can provide a more complete picture of syphilis acquisition and transmission dynamics within communities. Understanding the potential pathways for syphilis transmission is critical for addressing rising syphilis rates among MSM and developing network-informed behavioral interventions aimed at syphilis control.

## Abbreviations

CAB: community advisory board
CDC: Centers for Disease Control and Prevention
DSTDP: Division of Sexually Transmitted Disease Prevention
EMA: ecological momentary assessment
IRB: institutional review board
IWTK: I Want the Kit
MSM: men who have sex with men
MTL: Molecular Testing Labs
NEST: Network Epidemiology of Syphilis Transmission
P&S: primary and secondary
PrEP: pre-exposure prophylaxis
RDS: respondent-driven sampling
REDCap: Research Electronic Data Capture
STI: sexually transmitted infection
USHINE: Understanding Sexual Health in Networks

## Data Availability

The data sets generated during or analyzed during this study are not publicly available because the data are owned by each of the institutions affiliated with this project in Baltimore, Maryland; Chicago, Illinois; and Columbus, Ohio. Persons interested in analyzing these data should email the site of interest to inquire about data access.

## References

[1] Sexually transmitted disease surveillance 2020. Centers for Disease Control and Prevention.

[2] Peterman TA, Su J, Bernstein KT, Weinstock H (2015). Syphilis in the United States: on the rise?. Expert Rev Anti Infect Ther.

[3] Grey J, Torrone E, Weinstock H (2019). P528 Rates of primary and secondary syphilis among men who have sex with men by HIV status – 24 states, 2011–2015. Sexually Transmitted Infection.

[4] de Voux A, Kidd S, Grey JA, Rosenberg ES, Gift TL, Weinstock H, Bernstein KT (2017). State-specific rates of primary and secondary syphilis among men who have sex with men - United States, 2015. MMWR Morb Mortal Wkly Rep.

[5] Sexually Transmitted Disease Surveillance 2020. Centers for Disease Control and Prevention.

[6] Jarzebowski W, Caumes E, Dupin N, Farhi D, Lascaux A-S, Piketty C, de Truchis P, Bouldouyre M-A, Derradji O, Pacanowski J, Costagliola D, Grabar S, FHDH-ANRS CO4 Study Team (2012). Effect of early syphilis infection on plasma viral load and CD4 cell count in human immunodeficiency virus-infected men: results from the FHDH-ANRS CO4 cohort. Arch Intern Med.

[7] Buchacz K, Patel P, Taylor M, Kerndt PR, Byers RH, Holmberg SD, Klausner JD (2004). Syphilis increases HIV viral load and decreases CD4 cell counts in HIV-infected patients with new syphilis infections. AIDS.

[8] Fleming DT, Wasserheit JN (1999). From epidemiological synergy to public health policy and practice: the contribution of other sexually transmitted diseases to sexual transmission of HIV infection. Sex Transm Infect.

[9] Lynn W, Lightman S (2004). Syphilis and HIV: a dangerous combination. Lancet Infectious Diseases.

[10] Jennings JM, Tilchin C, Meza B, Schumacher C, Fields E, Latkin C, Rompalo A, Greenbaum A, Ghanem KG (2020). Overlapping transmission networks of early syphilis and/or newly hiv diagnosed gay, bisexual and other men who have sex with men (MSM): opportunities for optimizing public health interventions. AIDS Behav.

[11] Mayer KH (2018). Old pathogen, new challenges: a narrative review of the multilevel drivers of syphilis increasing in American men who have sex with men. Sexual Trans Dis.

[12] Morris M (2001). Concurrent partnerships and syphilis persistence: new thoughts on an old puzzle. Sex Transm Dis.

[13] Schneider JA, Cornwell B, Ostrow D, Michaels S, Schumm P, Laumann EO, Friedman S (2013). Network mixing and network influences most linked to HIV infection and risk behavior in the HIV epidemic among black men who have sex with men. Am J Public Health.

[14] Glick S, Morris M, Foxman B, Aral SO, Manhart LE, Holmes KK, Golden MR (2012). A comparison of sexual behavior patterns among men who have sex with men and heterosexual men and women. J Acquir Immune Defic Syndr.

[15] Tilchin C, Wagner J, Schumacher CM, Ghanem KG, Hamill MM, Rompalo A, Fields E, Latkin CA, Greenbaum A, Jennings JM (2022). HIV transmission potential and sex partner concurrency: evidence for racial disparities in HIV risk among gay and bisexual men (MSM). AIDS Behav.

[16] Jennings J, Ellen J, Deeds B, Harris DR, Muenz LR, Barnes W, Lee SS, Auerswald CL, Adolescent Trials Network for HIV/AIDS Interventions (2009). Youth living with HIV and partner-specific risk for the secondary transmission of HIV. Sex Transm Dis.

[17] Morris M, Kretzschmar M (1997). Concurrent partnerships and the spread of HIV. AIDS.

[18] Hamilton DT, Morris M (2015). The racial disparities in STI in the U.S.: concurrency, STI prevalence, and heterogeneity in partner selection. Epidemics.

[19] Koumans EH, Farley TA, Gibson JJ, Langley C, Ross MW, McFarlane M, Braxton J, St Louis ME (2001). Characteristics of persons with syphilis in areas of persisting syphilis in the United States: sustained transmission associated with concurrent partnerships. Sex Transm Dis.

[20] Traeger MW, Schroeder SE, Wright EJ, Hellard ME, Cornelisse VJ, Doyle JS, Stoové MA (2018). Effects of pre-exposure prophylaxis for the prevention of human immunodeficiency virus infection on sexual risk behavior in men who have sex with men: a systematic review and meta-analysis. Clin Infect Dis.

[21] Khosropour C, Dombrowski J, Kerani R, Katz D, Barbee L, Golden M (2016). Changes in condomless sex and serosorting among men who have sex with men after HIV diagnosis. J Acquir Immune Defic Syndr.

[22] Harawa N, Holloway I, Leibowitz A, Weiss R, Gildner J, Landovitz RJ, Perez MJ, Kulkarni S, Rotheram-Borus MJ, Shoptaw S (2017). Serious concerns regarding a meta-analysis of preexposure prophylaxis use and STI acquisition. AIDS.

[23] Mustanski B, Birkett M, Kuhns LM, Latkin CA, Muth SQ (2015). The role of geographic and network factors in racial disparities in HIV among young men who have sex with men: an egocentric network study. AIDS Behav.

[24] Raymond HF, McFarland W (2009). Racial mixing and HIV risk among men who have sex with men. AIDS Behav.

[25] Bonett S, Meanley S, Stevens R, Brawner B, Bauermeister J (2020). The role of networks in racial disparities in HIV incidence among men who have sex with men in the United States. AIDS Behav.

[26] Oster A, Wiegand R, Sionean C, Miles IJ, Thomas PE, Melendez-Morales L, Le BC, Millett GA (2011). Understanding disparities in HIV infection between black and white MSM in the United States. AIDS.

[27] Sullivan PS, Purcell DW, Grey JA, Bernstein KT, Gift TL, Wimbly TA, Hall E, Rosenberg ES (2018). Patterns of racial/ethnic disparities and prevalence in HIV and syphilis diagnoses among men who have sex with men, 2016: a novel data visualization. Am J Public Health.

[28] Laumann EO, Youm Y (1999). Racial/ethnic group differences in the prevalence of sexually transmitted diseases in the United States: a network explanation. Sex Transm Dis.

[29] Aral SO (1996). The social context of syphilis persistence in the southeastern United States. Sex Transm Dis.

[30] Chesson H, Kent C, Owusu-Edusei KJ, Leichliter J, Aral S (2012). Disparities in sexually transmitted disease rates across the "eight Americas". Sex Transm Dis.

[31] Hogben M, Leichliter J, Aral SO (2020). An overview of social and behavioral determinants of STI. Sexually Transmitted Infections.

[32] Juher D, Saldaña J, Kohn R, Bernstein K, Scoglio C (2017). Network-centric interventions to contain the syphilis epidemic in San Francisco. Sci Rep.

[33] Pathela P, Braunstein S, Schillinger J, Shepard C, Sweeney M, Blank S (2011). Men who have sex with men have a 140-fold higher risk for newly diagnosed HIV and syphilis compared with heterosexual men in New York City. J Acquir Immune Defic Syndr.

[34] Young LE, Fujimoto K (2021). Corrigendum to "The co-evolution of online social networks and syphilis incidence among young black men who have sex with men" [soc. Sci. Med. 272 (2020) 113764]. Soc Sci Med.

[35] Dauria E, Elifson K, Arriola K, Wingood G, Cooper H (2015). Male incarceration rates and rates of sexually transmitted infections: results from a longitudinal analysis in a southeastern US city. Sex Transm Dis.

[36] Chapin-Bardales J, Rosenberg E, Sullivan P, Jenness S, Paz-Bailey G, NHBS Study Group (2019). Trends in number and composition of sex partners among men who have sex with men in the United States, national HIV behavioral surveillance, 2008-2014. J Acquir Immune Defic Syndr.

[37] Cope AB, Bernstein K, Matthias J, Rahman M, Diesel J, Pugsley RA, Schillinger JA, Ng RA, Sachdev D, Shaw R, Nguyen TQ, Klingler EJ, Mobley VL, Samoff E, Peterman TA (2020). Unnamed partners from syphilis partner services interviews, 7 jurisdictions. Sex Transm Dis.

[38] Schumacher CM, Fields E, Chandran A, Heidari O, Kingon Y, Chaulk P, Jennings JM (2018). Investigation of early syphilis trends among men who have sex with men to identify gaps in screening and case-finding in Baltimore city, Maryland. Sex Transm Dis.

[39] (2017). HIV/STI Surveillance Report. Chicago Department of Public Health.

[40] (2019). Sexually transmitted infections. The City of Columbus.

[41] (2016). Syphilis data brief. Columbus Public Health.

[42] First year geographic focus ending the HIV epidemic: a plan for America. Centers for Disease Control and Prevention.

[43] Grieb SM, Jackman K, Tilchin C, Clark C, Sawyer S, Rives S, Childs L, Jennings JM, USHINE Community Advisory Board (2021). Recommendations from black sexual minority men: building trust to improve engagement and impact of HIV/STI research. Health Promot Pract.

[44] Ricks J, Spahnie M, Conroy S (2019). P553 Initiating a sexual network study among men who have sex with men: a mixed-methods pilot study. Sexually Transm Inf.

[45] Kuhns L, Birkett M, Muth SQ, Latkin C, Ortiz-Estes I, Garofalo R, Mustanski B (2015). Methods for collection of participant-aided sociograms for the study of social, sexual and substance-using networks among young men who have sex with men. Connect (Tor).

[46] Sheck I, Tilchin C, Wagner J, Epstein DH, Burgess-Hull A, Jennings JM (2022). Acceptability and feasibility of geographically explicit ecological momentary assessment among men who have sex with men. Arch Sex Behav.

[47] Harris PA, Taylor R, Thielke R, Payne J, Gonzalez N, Conde JG (2009). Research electronic data capture (REDCap)--a metadata-driven methodology and workflow process for providing translational research informatics support. J Biomed Inform.

[48] Harris PA, Taylor R, Minor BL, Elliott V, Fernandez M, O'Neal L, McLeod L, Delacqua G, Delacqua F, Kirby J, Duda SN, REDCap Consortium (2019). The REDCap consortium: building an international community of software platform partners. J Biomed Inform.

[49] I want the Kit homepage. I Want the Kit.

[50] Hogenson E, Jett-Goheen M, Gaydos CA (2019). An analysis of user survey data for an internet program for testing for sexually transmitted infections, I want the kit, in Maryland and Washington, DC. Sex Transm Dis.

[51] Norelli J, Zlotorzynska M, Sanchez T, Sullivan PS (2021). Scaling up CareKit: lessons learned from expansion of a centralized home HIV and sexually transmitted infection testing program. Sex Transm Dis.

[52] Everett BG (2013). Sexual orientation disparities in sexually transmitted infections: examining the intersection between sexual identity and sexual behavior. Arch Sex Behav.

[53] Palar K, Laraia B, Tsai A, Johnson M, Weiser S (2016). Food insecurity is associated with HIV, sexually transmitted infections and drug use among men in the United States. AIDS.

[54] Williams SP, Bryant KL (2018). Sexually transmitted infection prevalence among homeless adults in the United States: a systematic literature review. Sex Transm Dis.

[55] Jennings JM, Wagner J, Tilchin C, Schumacher CM, Thornton N, Hamill MM, Rompalo A, Ruhs S, Rives S, Ghanem KG, Latkin C (2021). Methamphetamine use, syphilis, and specific online sex partner meeting venues are associated with HIV status among urban black gay and bisexual men who have sex men. Sex Transm Dis.

[56] Brantley M, Schumacher C, Fields EL, Perin J, Safi AG, Ellen JM, Muvva R, Chaulk P, Jennings JM (2017). The network structure of sex partner meeting places reported by HIV-infected MSM: opportunities for HIV targeted control. Soc Sci Med.

[57] Jennings J, Reilly M, Perin J, Schumacher C, Sharma M, Safi AG, Fields EL, Muvva R, Nganga-Good C, Chaulk P (2015). Sex partner meeting places over time among newly HIV-diagnosed men who have sex with men in Baltimore, Maryland. Sex Transm Dis.

[58] Tourangeau R, Yan T (2007). Sensitive questions in surveys. Psychol Bull.

[59] Chandra C, Morris M, Van Meter C, Goodreau SM, Sanchez T, Janulis P, Birkett M, Jenness SM (2022). Comparing sexual network mean active degree measurement metrics among men who have sex with men. Sex Transm Dis.

[60] Uong S, Rosenberg E, Goodreau S, Luisi N, Sullivan P, Jenness S (2020). Assessment of bias in estimates of sexual network degree using prospective cohort data. Epidemiology.

[61] Weiss KM, Goodreau SM, Morris M, Prasad P, Ramaraju R, Sanchez T, Jenness SM (2020). Egocentric sexual networks of men who have sex with men in the United States: results from the ARTnet study. Epidemics.

[62] (2009). Consultation on concurrent sexual partnerships. UNAIDS.

[63] Hood JE, Friedman AL (2011). Unveiling the hidden epidemic: a review of stigma associated with sexually transmissible infections. Sex Health.

[64] Fields EL, Long A, Silvestri F, Bademosi K, Benton-Denny J, Granderson R, Schumacher C, Chandran A, Greenbaum A, Jennings J (2022). #ProjectPresence: highlighting black LGBTQ persons and communities to reduce stigma: a program evaluation. Eval Program Plann.

[65] Alsan M, Wanamaker M, Hardeman RR (2020). The tuskegee study of untreated syphilis: a case study in peripheral trauma with implications for health professionals. J Gen Intern Med.

[66] Plant A, Stahlman S, Javanbakht M, Cross J, Montoya JA, Bolan R, Kerndt PR (2015). Syphilis experiences and risk perceptions among repeatedly infected men who have sex with men. Perspect Sex Reprod Health.

[67] Sawyer S, Grieb SM, Long A, Tilchin C, Clark C, Greenbaum A, Jennings JM, USHINE Community Advisory Board (2021). Improving research dissemination to Black sexual minority men: development of a community-led and theory-based dissemination plan. Health Promot Pract.

